# Editorial: Management of training frequency and intersession recovery—Molecular, cellular and physiological implications for performance and health

**DOI:** 10.3389/fphys.2022.1093532

**Published:** 2022-12-01

**Authors:** Jorming Goh, Yifan Yang, Peter Hofmann, Chin Leong Lim

**Affiliations:** ^1^ Healthy Longevity Translational Research Programme, Yong Loo Lin School of Medicine, National University of Singapore (NUS), Singapore, Singapore; ^2^ Department of Physiology, Yong Loo Lin School of Medicine, NUS, Singapore, Singapore; ^3^ Centre for Healthy Longevity, National University Health Systems (NUHS), Singapore, Singapore; ^4^ Office of Education Research, National Institute of Education, Nanyang Technological University, Singapore, Singapore; ^5^ Exercise Physiology, Training and Training Therapy Research Group, Institute of Human Movement Science, Sport and Health, University of Graz, Graz, Austria; ^6^ Lee Kong Chian School of Medicine, Nanyang Technological University, Singapore, Singapore

**Keywords:** exercise, recovery, molecular, training load, physiological

## Introduction

While scientific knowledge in the performance and training aspects of exercise physiology has grown in the past few decades, especially in the application of molecular and cellular techniques to interrogate mechanistic pathways, similar progress has not been achieved in elucidating the science of recovery from exercise training. In elite and recreational sports, there has been a worldwide explosion of interest in marathons, ultra-endurance races, as well as high intensity interval training (HITT) programs implemented across a wide variety of settings. These events involve large numbers of participants, meaning that individuals now have access to, and may be performing significantly higher amounts of exercise than may be optimal for their health. In addition, there has been growing acceptance for exercise training as concurrent treatment programs for clinical disease, such as for cancer. In such clinical populations, the duration of recovery between exercise sessions could be very different from healthy populations. This special collection called for submissions investigating physiological responses during recovery or rest phases. Such rest phases are usually contextualized within athletic training periodization, where an entire season of training is termed the *macrocycle*, followed by blocks of *mesocycles*—typically lasting weeks, and finally the *microcycle*, which is a typical week of training. While these cycles are usually planned with elite athletic performance in mind, they can also be incorporated into the clinical setting, for instance to monitor athlete or patient response after injury or illness. Although similar conceptually, the design of the periodization (training load and recovery ratio) and mix of training variables (frequency, intensity and duration) would be very different between athlete and diseased population.

In the first submission, (Allan et al.), raised an important concept for exercise training in cancer patients, which is the often-underappreciated metabolic stress of the disease, particularly for those with advanced disease and experiencing tissue cachexia. The authors contend that the additional metabolic stressor may counteract some beneficial adaptations of exercise, given a continuous tissue breakdown, increased systemic and tissue inflammation, as well as the increased energetic needs for anti-tumor immunity. This situation thus results in the competition between the tumor and the host for metabolic resources, ultimately compromising potential benefits that could have been gleaned from exercise training. The authors suggest that exercise programs prescribed for cancer patients should be lower in terms of volume/intensity and furthermore, to allow sufficient time for recovery, meaning that the frequency of exercise sessions must be tapered down and personalized for each individual.

Having the appropriate mix between training load and recovery sets the foundation for positive adaptation in physical and sport training and for lowering the risk of overtraining. While external training load (actual physical work performed) is explicit and can be quantified accurately (e.g., frequency, speed and duration), accurate quantification of internal training load (e.g., physiological stress and responses) is more challenging, especially in field settings. Accurate qualification and matching of the internal and external training loads can potentially offer advantages in optimizing training adaptation and in personalizing training program design. The measurement heart rate and rating of perceived exertion (RPE) have been time-tested tools for quantifying internal training load in both research and sport training. However, fundamental gaps exist in their sensitivity to accurately reflect internal training load when used alone, indifferent forms, or in combination with other parameters.

Pind and others (Pind et al.) investigated these classical issues of sport science and training in their study on elite rowers undergoing 4 weeks of training camp. The elegance of this study is found in the fine dissection of HR and RPE, in different forms and combination, to track the dynamics of the external training load over 4 weeks. A key finding from this study is that as training intensity increased, RPE had a stronger association with external training load than heart rate, and the state of fatigue was positively associated with RPE, but not with heart rate. These results support the notion that “internal” adaptations to training stresses is due to the sum of both psychological and physiological responses, which is better sensed by RPE than by heart rate. This study should also arouse the curiosity of researchers and practitioners on the potential cross-talks between the psychological and physiological systems in regulating training response and adaptation i.e., human performance as a whole being.

In “Electrical and Structural Adaption Athlete’s Heart and the Impact on Training and Recovery Management in Professional Basketball Players Retrospective Observational Study”, (Zimmermann et al.), assessed the electrocardiogram (ECG) analyses, transthoracic echocardiographic examinations and cardiopulmonary exercise testing (CPET) of 27 young male professional basketball players. The cohort was segregated retrospectively into 12 with early polarization (ER) pattern and 15 without ER pattern. Once thought to be benign, there have been higher associations of cardiac events or abnormalities in endurance trained, and non-athletic populations with ER pattern. This study addressed a gap by studying professional basketball players. The authors found that professional male basketball players with an ER pattern had higher absolute and relative peak oxygen uptakes in CPET compared to those without ER pattern, concurring with the common ER phenomenon in athletes and that increased exercise training is associated with a greater occurrence of ER pattern. The professional basketball players with ER patterns also had larger left ventricular end-diastolic diameter, left and right atrial end systolic diameters, and left ventricular mass Index without significant differences in relative wall thickness and ECG parameters at rest and during exercise testing. These findings contribute towards our understanding of cardiac remodeling in athletes with ER pattern. Specific athletes present with abnormalities such as early repolarization, which may suggest a greater risk for atrial fibrillation and sudden cardiac death, even during early pre-season, when they are engaging in low-intensity and high-volume training, This study highlights the need for echocardiographic assessment of professional basketball players as part of individualized prescription, to monitor cardiac remodeling throughout the season.

Usually, the main focus of exercise prescription is on intensity, which however, is just one part of the F.I.T.T. (T.) principle (Burnet et al., 2019). This principle states that beside Intensity, Frequency, Time (duration) and Type as well as Timing (Reid et al., 2019) play an additional role. Unfortunately, most exercise studies do not consider and present all of these key parameters of exercise prescription (Campbell et al., 2012; Winters-Stone et al., 2014; Neil-Sztramko et al., 2019). It is therefore interesting to see new articles investigating variations of some of these rarely investigated variables such as in the manuscript by (Senna et al.) regarding “Higher muscle damage triggered by shorter inter-set rest periods in volume equated resistance exercise”. This article nicely shows the impact of rest period variations on inflammatory responses and muscle damage due to a small variation of recovery time between five sets of 10 reps resistance exercise performed at the same volume and at the same intensity of the 10RM. One- or 3-min rest periods significantly influenced circulating concentrations of selected biomarkers. Shorter recovery between sets produced greater increases in selected variables post exercise compared to longer recovery. The authors concluded that a 1-min rest condition in volume equated resistance exercise promoted greater overall muscle tissue damage with a longer duration of the 24 inflammatory processes compared to the 3-min rest conditions. These results clearly highlight the need to consider additional variables of training load beside intensity and volume of exercise such as highlighted recently (Neil-Sztramko et al., 2019).

## Conclusion

The articles presented in this special collection have addressed the science of rest or recovery from exercise training across the clinical, recreational and professional exercise settings. In this regard, recovery needs to be personalized to achieve the greatest benefits from training for both health and performance. There is much still unknown in terms of recovery optimization and should be a focus for future research.

## References

[B1] Burnet K. Kelsch E. Zieff G. Moore J. B. Stoner L. (2019). How fitting is F.I.T.T.? A perspective on a transition from the sole use of frequency, intensity, time, and type in exercise prescription. Physiol. Behav. 199, 33–34. 10.1016/j.physbeh.2018.11.007 30414400

[B2] Campbell K. L. Neil S. E. Winters-Stone K. M. (2012). Review of exercise studies in breast cancer survivors: Attention to principles of exercise training. Br. J. Sports Med. 46, 909–916. 10.1136/bjsports-2010-082719 23007178

[B3] Neil-Sztramko S. E. Winters-Stone K. M. Bland K. A. Campbell K. L. (2019). Updated systematic review of exercise studies in breast cancer survivors: Attention to the principles of exercise training. Br. J. Sports Med. 53, 504–512. 10.1136/bjsports-2017-098389 29162619

[B4] Reid R. E. R. Thivel D. Mathieu M. E. (2019). Understanding the potential contribution of a third "T" to FITT exercise prescription: The case of timing in exercise for obesity and cardiometabolic management in children. Appl. Physiol. Nutr. Metab. 44, 911–914. 10.1139/apnm-2018-0462 30875478

[B5] Winters-Stone K. M. Neil S. E. Campbell K. L. (2014). Attention to principles of exercise training: A review of exercise studies for survivors of cancers other than breast. Br. J. Sports Med. 48, 987–995. 10.1136/bjsports-2012-091732 23293010

